# Supplementary material for: β-Glucocerebrosidase Deficiency Activates an Aberrant Lysosome-Plasma Membrane Axis Responsible for the Onset of Neurodegeneration
Source: Cells. 2022 Jul 29;11(15):2343. doi: 10.3390/cells11152343 (PMC9367513; doi:10.3390/cells11152343)
Supplement: Supplementary file 1 [file cells-11-02343-s001.zip › cells-1804865-supplementary/cells-1804865-supplementary.pdf]

Supporting information

β-glucocerebrosidase deficiency activates an aberrant lysosome-plasma membrane axis responsible for the onset of neurodegeneration

Giulia Lunghi[1*], Emma Veronica Carsana[1*], Nicoletta Loberto[1], Laura Cioccarelli[1], Simona Prioni[1], Laura Mauri[1], Rosaria Bassi[1], Stefano Duga[2,3], Letizia Straniero[2,3], Rosanna Asselta[2,3], Giulia Soldà[2,3], Alessio Di Fonzo[4], Emanuele Frattini[4], Nara Liessi[5], Andrea Armirotti[5], Elena Ferrari[6], Maura Samarani[7 †], Massimo Aureli[1†]

[1]Department of Medical Biotechnology and Translational Medicine, University of Milan, Milan, Italy
[2]Department of Biomedical Sciences, Humanitas University, Via Rita Levi Montalcini 4, 20090 Pieve Emanuele, Milan, Italy
[3]Humanitas Clinical and Research Center, IRCCS, Via Manzoni 56, 20072 Rozzano, Milan, Italy
[4]IRCCS Foundation Ca' Granda Ospedale Maggiore Policlinico, Dino Ferrari Center, Neuroscience Section, Department of Pathophysiology and Transplantation, University of Milan, Milan, Italy
[5] Analytical Chemistry Facility, Fondazione Istituto Italiano di Tecnologia, Via Morego 30, 16163, Genoa, Italy
[6]Department of Pharmacological and Biomolecular Sciences, University of Milan, Milan, Italy
[6]Department of Cell Biology and Infection, Institut Pasteur, Paris, France

* These authors equally contributed
† These authors share the senior position

Corresponding author:
Massimo Aureli, Department of Medical Biotechnology and Translational Medicine, University of Milan, Milan, Italy, Tel: 0250330364, massimo.aureli@unimi.it

Methods

Glucosylsphingosine treatment

Glucosylsphingosine (GlcSph) was solubilized directly in the culture medium of CGN and DA neurons at 100 nM and 10 μM (final concentration) of and administered to CGN at 14 DIC for 24 h. Untreated cells were incubated under the same experimental conditions without GlcSph.

Calcein assay

Cells were plated in 96-well microplate at a density of 3.15 x 10^5 cells/cm^2 for CGN and 2 x 10^5 cells/cm^2 for DA neurons. After the removal of culture medium, cells were washed with PBS and then incubated with 5 μg/ ml Calcein-AM solution in of PBS (15 min, 37°C). At the end of incubation, Calcein-AM solution was discarded and cells were lysed with 1% Triton X-100 in TNEV buffer in mild agitation (10 min, 23°C). The cell lysates were transferred to a black microplate (Black, 96-well, OptiPlate-96 F, Perkin Elmer) and fluorescence intensity (ex/em 495/515 nm) was measured by a Victor microplate reader (Perkin Elmer).

MTT assay

CGNs and DA neurons were plated in 96-well microplate at a density of 3.15 x 10^5 cells/cm^2 and 2 x 10^5 cells/cm^2 respectively. Cells were incubated in culture medium containing 1 mg/ml MTT at 37°C. After 2 hours, MTT solution was discarded and the blue MTT–formazan product was extracted with 2-propanol: formic acid, 95: 5 (v:v). After 15 min of gentle agitation at room temperature, the absorbance of the formazan solution was read at 570 nm by a microplate spectrophotometer (Wallac 1420 VICTOR2 TM; Perkin Elmer).

Mass spectrometry and data analysis

The analysis of GlcCer content was carried out on aliquots of the organic phase, subjected to alkaline treatment to remove glycerophospholipids[1]. The analysis of GlcSph content was carried out as previously described[2]. Lyophilized cell lysates were extracted with acetone, the extracts were dried under nitrogen flow and then resuspended in chloroform: methanol 2: 1 (v: v). GlcCer and GlcSph were analysed by LC ESI-MS/MS according to Merrill, A. H., Jr., 2011[3] with proper modifications[4]. Mass spectrometry analyses were carried out using LCQDeca ion trap mass spectrometer (Thermo Fisher Scientific) equipped with an electrospray ionization source, an Xcalibur data system and a Jasco PU980 Pump HPLC. GlcCer and GlcSph were separated and detected using an HPLC system (JASCO; Tokyo, Japan) equipped with a normal phase column LC-Si (Supelco 2.1 mm x 250 mm x 5um). Elution was carried out at a flow rate of 0.700 ml/min, with a gradient formed by solvent system A: CH_3CN: CH_3OH: H_3CCOOH 97: 2: 1 (v: v: v) with 5 mM ammonium acetate and solvent system B: CH_3OH containing 5 mM ammonium acetate. MS analyses were carried out at a positive ion mode MS/MS, sheath gas flow of 60 arbitrary units, spray voltage of 5 kV, capillary voltage of 70 eV and capillary temperature of 300°C. Mass spectra were acquired over a range of m/z 200–2 000.

Immunofluorescence staining

DA neurons were plated at a density of 10^5 cells/cm^2 on 24 mm cover glasses precoated with geltrex (1%, 1 h 37°C). Neurons were fixed in 4% paraformaldehyde in PBS for 20 min at 23°C. Cells were then permeabilized in 0.2 % Triton X-100 in PBS for 10 min at 23°C and blocked in 1% BSA/ 2% Donkey serum in PBS for 1 h at 23°C. Cells were incubated 2 h at 23°C with primary antibodies diluted in 0.25% BSA/ 0.5% Donkey serum in PBS and 1 h at 23°C with secondary antibodies conjugated to Alexa Fluor, diluted in the same solution. Nuclei were stained by incubation with Hoechst solution (2 µg/ml in PBS) for 5 min at 23°C. The following antibodies were used: monoclonal mouse anti-β3-tubulin (4466; CellSignaling Technology; dilution: 1:200), monoclonal mouse anti-TH (F-11) (25269; SantaCruz; dilution: 1:50), mouse anti-LAMP1 (AB_2296838; dilution: 1:50; DSHB), polyclonal anti-mouse AlexaFluor488 (A21202; ThermoFisher Scientific) and anti-mouse AlexaFluor568 (A10037; ThermoFisher Scientific). Coverslips were mounted with Dako Fluorescent

Mounting Medium (Agilent Technologies, Santa Cruz, CA, USA). Images were taken using an Olympus BX50 Upright Fluorescence Microscope fluorescence with a fast-high resolution camera (Colorview 12).

Analysis of endogenous lipid pattern by HPTLC

Lipids were extracted from CBE-treated and untreated CGNs and DA neurons and subjected to two-phase partitioning, as described in the Methods section.

For each sample, equal amounts in terms of cell proteins were loaded on HPTLC and lipids were separated using the solvent system chloroform: methanol: H_2O 110:40:6 (v:v:v).

Endogenous lipid content was detected by spraying the HPTLC with anisaldehyde reagent. Identification of lipids after separation was assessed by comigration with authentic standards. The relative amounts of lipids were determined by densitometry analysis using ImageJ software.

Lysotracker staining

CGNs were plated in 24-well plate at a density of 3.15 x 10^5 cells/cm^2 and were treated or not with CBE for 17 days. At 20 DIC LysoTracker Red DND-99 (Molecular Probes) was directly diluted in the culture medium at 50 nM final concentration (30 minutes, 37°C, 5% CO_2). Cells were then washed with PBS and images were acquired with Olympus IX50 Inverted Fluorescence Microscope equipped with a halogen lamp directly on live cells at 100x magnification.

Transmission electron microscopy

Cell monolayers were fixed in 2.5% glutaraldehyde, 0.1 M sodium cacodylate buffer pH 7.4 for 1 h at 23°C, washed three times with cacodylate buffer and post-fixed with 1% osmium tetroxide,1.5% potassium ferrocyanide in 0.1 M cacodylate for 1 h on ice.

After several washes in distilled water, samples were "en bloc" stained with 0.5% uranyl acetate in water overnight at 4°C. Samples were dehydrated in a graded ethanol series (30%, 50%,70%, 80%,90%,96%, 5 minutes each followed by 3 washes with absolute ethanol, 10 min each). The

samples were then infiltrated in a 1:1 ethanol: Epon 812 solution for 2 hours and in 100% Epon twice for 1 h each. The Epon-embedded cell monolayers were then polymerized in an oven at 60°C for 48 hours. At the end of the polymerization the plastic was separated from the Epon block mechanically breaking the plastic, leaving the cell monolayer facing up on the resin block.

A portion of the specimen was glued on top of an Epon block and mounted on a Leica Ultracut UCT ultramicrotome. Ultrathin (70-90nm) sections were then collected on copper grids and stained with uranyl acetate and lead citrate. Grids were examined with a transmission electron microscope (TALOS L120C ThermoScientific) at 120 kv.

Treatment of cell cultures with [3-^{3}H(sphingosine)] -GM1

Isotopically labeled [3-^{3}H(sphingosine)]-GM1 dissolved in propanol: H_2O 7:3 (v:v) was transferred into a sterile glass tube, dried under a nitrogen stream, solubilized in an appropriate volume of culture medium (0,067 µCi /ml of medium) and administered to cells. After 4 h, cells were harvested with PBS and processed for lipid extraction and analysis as already described. TLE were then separated by HPTLC (MilliporeSigma) with the solvent system chloroform: methanol: $CaCl_2$ (50: 42: 11 v: v: v). Radioactive lipids were detected by digital autoradiography (^{T}Racer betaimager; BioSpace Laboratory) and quantified using M3 Vision software. Lipids were identified after separation by comigration with radioactive lipid standards.

Immunoblotting analysis of SN and P fractions after cell surface biotinylation

Proportional amounts of SN and P fractions from CGN or DA neurons were separated on 4–20% polyacrylamide gradient gel and transferred to PVDF membranes by electroblotting. Membranes were blocked in 5 % non-fat dry milk solution. Subsequently PVDFs were incubated overnight at 4°C with the primary antibody and secondary HRP-conjugated antibodies in 5 % non-fat dry milk solution for 1 h at 23°C. Biotinylated proteins were detected with HRP-conjugated streptavidin using the chemiluminescent system Alliance Mini HD9 (Uvitec, Cambridge, United Kingdom). Band intensity was quantified using ImageJ software (v2.1.0/1.53c). The following primary antibodies were used for

immunoblotting: Polyclonal rabbit anti-PSD95 (25072; Cell Signaling Tecnhology; dilution: 1:1 000), polyclonal rabbit anti-GAPDH (G9535, MilliporeSigma; dilution: 1: 7 000).

The following secondary antibodies were used: Goat-anti-rabbit HRP-conjugated (7074; CellSignaling Technology; 1: 2 000) and Goat-anti-mouse HRP conjugated (31430; ThermoFisher; dilution: 1: 5 000) Streptavidin HRP-conjugated (HRP; SA-5004; Vector Laboratories, Burlingame, CA, USA).

Immunofluorescence of iPSCs

iPSCs were grown on suitable culture imaging plates to allow visualization of the cells following immunofluorescence by inverted wide field and confocal microscopes. Cells were fixed with 4% paraformaldehyde (PFA) for 30 minutes. Then cells were rinsed twice with PBS 1X, permeabilized and quenched with wash-buffer (PBS 1X + BSA 3% + Goat serum (Vector) 5% + Triton X-100 (Euroclone) 0.3%), blocked for 30 minutes at room temperature (RT). Plates were subsequently incubated with primary antibodies overnight at 4°C (for primary antibodies dilution, see antibody Table), washed three times in wash buffer (PBS + 0.1% Triton X-100) for 5 minutes each, then incubated with secondary antibodies (Alexa 488, 568, 647, Molecular Probes; 1:500) and DAPI (1:1000) nuclear counterstain for 10 minutes at room temperature, and finally washed as previously described. Plates were then mounted on imaging slides (Thermo Fisher Scientific). Cells were stored at 4°C in dark until image acquisition through confocal microscope.

Antibody table

Primary Antibody	Host	Vendor	Dilution
OCT4	Rabbit	Cell Signaling	1:100
SSEA4	Mouse	Cell Signaling	1:200
SOX2	Rabbit	Cell Signaling	1:200
NANOG	Rabbit	Cell Signaling	1:200

TRA160	Mouse	Cell Signaling	1:500
TRA181	Mouse	Cell Signaling	1:500

Real-time PCR (qPCR)

RNA extraction

RNA extraction was achieved by TRIzol^{TM} Reagent (Invitrogen). After a mechanical homogenization of the sample with 1 mL of TRIzol^{TM} Reagent, 200 µL of ice-cold chloroform (Sigma-Aldrich) was added. Samples were vortexed until the two phases mixed together to form an emulsion. After that, samples were centrifuged for 10 min at 4°C at 13’500 rpm. RNA phase was collected in a new tube and ice-cold isopropanol (Sigma-Aldrich) was added in 1:1 ratio, mixing gently. The RNA was precipitated overnight at -20°C. Samples were then centrifuged for 20 minutes at 4°C at 13,500 rpm. RNA pellets were then washed two or three times with 1 mL of ice-cold 70% ethanol (Sigma-Aldrich). For every wash, samples were centrifuged for 5 minutes at 6,000 rpm at 4°C. After the last one, RNA pellets were dried in fume hood for a few minutes until they become transparent. RNA pellets were resuspended in RNAse-free water based on the RNA pellet dimension (usually 20-30 µL for a medium-sized pellet) and kept at -80 degrees until further use.

Quantification of extracted RNA by UV absorption

RNA was quantified using UV absorption and the absorbance was measured at RNA’s maximum absorption wavelength (260 nm) through a spectrophotometer. The linear relationship between absorbance and concentration of an absorbing specie was determinate by the Beer-Lambert law, $A = \log (I0/I) = \varepsilon cl$, which predicts a linear change in absorbance with concentration. The NanoDrop 1000 Spectrophotometer (Thermo Fisher Scientific) was used to perform the analyses. The ratios of the absorbance 260/280nm (~2.0) and 260/230 (~1.8-2.2) were used to assess the RNA purity. RNA

samples were diluted to reach 500 ng/µL and after every dilution the RNA concentrations were quantified again.

RT-PCR: Retrotranscription of RNA into cDNA

The iScript cDNA Synthesis Kit (Bio-Rad) was used to perform the retrotranscription. This kit contains oligo (dT), random hexamer primers and a reverse transcriptase enzyme. To perform retrotranscription, 500 ng of RNA were transferred in a PCR RNAse-free tube and nuclease-free water was added up to 15 µL volume. Then 5 µL of mix solution (4 µL 5X iScript Reaction Mix/Buffer, 1 µL iScript Reverse Transcriptase) were added and the PCR tube containing complete reaction mix was incubated in a thermal cycler using the RT-PCR protocol. cDNA was stored at -20°C until use.

qPCR

The qPCR mix used was the SsoFast™ EvaGreen® Supermix 2X (Bio-Rad). Retrotranscribed cDNA from RT-PCR was further diluted (1:10) and 5 µL of diluted cDNA for each sample was loaded in triplicate in a Hard-Shell® 96-Well PCR Plates (Bio-Rad).

15 µL of SsoFast™ EvaGreen® Supermix diluted with nuclease-free water and with the specific primers (see primer Table) for the target of interest, were added. In this final qPCR reaction volume, Supermix reachs the right dilution and primers get 10 µM concentration. Routine qPCR negative controls were included in the plate and the qPCR protocol was performed using a CFX96™ Real-Time System (Bio-Rad) coupled with a C1000™ Thermal Cycler (Bio-Rad).

Once qPCR was carried out, data were analyzed with the CFX Manager Software (BioRad) and excel data sheet tool. Gene expression was achieved through the use of ΔCT as relative gene expression analysis using a reference gene. ΔCT was calculated through Ct normalization of the target gene to the reference YWHAZ gene.

$2^{(Ct(ref)-Ct(sample))}$ = Expression

Primer table

PRIMER	SEQUENCE 5'>3'
YWHAZ-FW	ACTTTTGGTACATTGTGGCTTCAA
YWHAZ-RV	CCGCCAGGACAAACCAGTAT
OCT4-FW	AGGCTCTGAGGTGTGGGGGAT
OCT4-RV	TGAGAGGTCTCCAAGCCGCCT
NANOG-FW	CATGAGTGTGGATCCAGCTTG
NANOG-RV	CCTGAATAAGCAGATCCATGG

Karyotype analysis

Following incubation with colchicine for 3 hours, iPSCs were incubated with 0.6% sodium citrate and 0.13% potassium chloride, fixed with methanol/acetic acid, and incubated with Quinacrine solution to obtain Q-banding. Acquired metaphases were analyzed with MetaSyste-Ikaros.

Statistical analyses

All statistical analyses were performed using GraphPad Prism 7 (GraphPad Software Inc., La Jolla, CA, USA). Data are expressed as mean ± SEM. For normally distributed data, two-tailed unpaired Student's *t* test, one-way ANOVA or two-way ANOVA tests were used. A p-value < 0.05 was considered significant.

Figure Legends

Supplementary Figure S1. Generation of in vitro neuronal models of β-glucocerebrosidase deficiency Schematic diagrams of the experimental protocols to induce β-glucocerebrosidase deficiency in two neuronal models: a primary cultures of granule neurons from the post-natal mouse cerebellum (CGN); b dopaminergic neurons from human-induced pluripotent stem cells (iPSCs). During differentiation of both the neuronal cultures, cell sphingolipids were metabolically labeled at the steady state with radioactive sphingosine; at the stage of mature neurons, cells were then treated with 0.5 mM conduritol B epoxide (CBE) to inhibit β-glucocerebrosidase. After different time points of CBE treatment, neurons were analyzed for β-glucocerebrosidase activity, neuronal marker expression and sphingolipid levels. DIC: days in culture; DOT: days of CBE treatment.

Supplementary Figure S2. Characterization of iPSCs. **a**. Representative immunofluorescence images of iPSC colonies showing expression of stem cell markers (OCT4, SSEA4, SOX2, TRA181, TRA160, and NANOG). Images were acquired at 20X magnification. **b**. qPCR showing expression levels of stem cell genes OCT4 and NANOG in iPSCs compared with fibroblasts. Data are shown as absolute normalized amount of mRNA (2-ΔCt) ± s.e.m. (iPSCs N=8, Fibroblasts N=2; ***P < 0.001, *P < 0.05, Student's *t*-test). **c**.Karyotype analysis of a selected clone of the generated iPSC line.

Supplementary Figure S3. Evaluation of differentiation of iPSC-derived dopaminergic neurons by immunofluorescence analysis. Representative immunofluorescence images of human iPSCs-derived dopaminergic neurons after 29 days of differentiation. Cells were positive for neuron-specific class III β-tubulin (TUJ1) and for the dopaminergic marker Tyrosine Hydroxylase (TH). Cell nuclei were stained with Hoechst. Images were acquired at 400x magnification.

Supplementary figure S4. Effect of CBE treatment on dopaminergic neurons differentiation. **a** Representative immunofluorescence images of human iPSCs-derived dopaminergic neurons treated or not with 0.5 mM CBE for 29 days. Cells were positive for the neuronal markers β-III-tubulin (TUJ1) and microtubule associated protein 2 (MAP2). Cell nuclei were stained with Hoechst. Images were acquired at 200x magnification. **b** Cell counting of dopaminergic neurons derived from human iPSCs treated or not (CTRL) with CBE 0.5 mM for 29 days. The graph shows the mean ± SEM of the fold change over control from three different experiments; **** p< 0.001, Student's t-test vs CBE-untreated cells.

Supplementary figure S5 Effect of GCase inhibition on GlcCer level. Representative HPTLC separation of endogenous lipids of the organic phases extracted from: **a** mouse cerebellar granule neurons and **b** human iPSCs-derived dopaminergic neurons treated or not with 0.5 mM CBE for 14 days and 29 days, respectively. Cer=ceramide; GlcCer= glucosylceramide; PE=phosphatidylethanolamine; Sulf=sulphatides; SM= sphingomyelin); GlcCer content was quantified by densitometric analysis; data are expressed as fold change with respect to CBE-untreated cells and are the mean ± SEM of three different experiments; ***$p<0.001$; ****$p<0.0001$. Student's t-test vs CBE-untreated cells.

Supplementary figure S6. HPLC elution profiles of total lipid extracts and ESI-MS analyses of glucosylceramide. **a** Representative chromatogram of the HPLC elution profile of total lipid extracts from mouse cerebellar granule neurons treated or not (CTRL) with 0.5 mM CBE; in grey is highlighted the peak corresponding to glucosylceramide (GlcCer) **b** Mass spectrum of GlcCer content of mouse cerebellar granule neurons treated or not (CTRL) with 0.5 mM CBE.

Supplementary figure S7 HPLC elution profiles of total lipid extracts and ESI-MS analyses of glucosylsphingosine. **a** Representative chromatogram of the HPLC elution profile of total lipid extracts from mouse cerebellar granule neurons treated or not (CTRL) with 0.5 mM conduritol B epoxide (CBE); in grey is highlighted the peak corresponding to glucosylsphingosine (GlcSph) **b** Mass spectrum of GlcSph content of mouse cerebellar granule neurons treated or not (CTRL) with 0.5 mM CBE.

Supplementary figure S8. Effect of GlcSph administration on cell viability. Glucosylsphingosine (GlcSph) was administered to **a** cerebellar granule neurons and **b** human iPSC-derived dopaminergic neurons at 0,1 and 10 µM for 24 hours. Cell viability was evaluated by Calcein assay and MTT assay. Data are expressed as fold change with respect to GlcSph-untreated cells (-) and are the mean ± SEM of at least three experiments; * $p < 0.05$, ** $p < 0.01$, *** $p < 0.001$, One-way ANOVA vs GlcSph-untreated cells.

Supplementary figure S9. Effect of β-glucocerebrosidase inhibition on ganglioside levels. Representative digital autoradiography of ganglioside pattern and quantification of the radioactivity associated with individual gangliosides in: **a** mouse cerebellar granule neurons fed with radioactive sphingosine and treated or not (CTRL) with 0.5 mM conduritol B epoxide (CBE) for 14 days, and **b** human iPSC-derived dopaminergic neurons fed with radioactive sphingosine and treated or not

(CTRL) with 0.5 mM CBE for 29 days. Data are expressed as fold change with respect to CBE-untreated cells (dashed line) and are the mean ± SEM of three different experiments. *p<0.05; **p<0.01; ***p<0.001, two-tail Student's t-test vs CBE-untreated cells.

Supplementary figure S10. Evaluation of TFEB nuclear translocation

Representative images of the expression of the transcription factor EB (TFEB) in the cytosolic and nuclear fraction of **a** mouse cerebellar granule neurons and **b** human iPSCs-derived dopaminergic neurons treated or not with 0.5 mM conduritol B epoxide (CBE). Cytosolic and nuclear fraction are represented respectively by the expression of the cytosolic marker GAPDH or the nuclear marker histone 3 (H3). Quantification of the nuclear amount of TFEB normalized on H3 and expressed as fold change with respect to CBE-untreataed cells (CTRL) and are the mean ± SEM of at least three experiments; * p < 0.05; ** p < 0.01, Student's t-test vs CBE-untreated cells.

Supplementary figure S11. Evaluation of lysosomal biogenesis upon glucosylceramide accumulation. **a** a Representative images of lysotracker Red DND-99 staining of mouse cerebellar granule neurons treated or not with 0.5 mM conduritol B epoxide (CBE) for 14 days. Images were acquired at 100x magnification and fluorescence intensity was quantified. b Expression of LAMP-1 in human iPSC-derived dopaminergic neurons treated or not with 0.5 mM CBE for 29 days, evaluated by immunofluorescence. Images were acquired at 400x magnification and fluorescence intensity was quantified. c Representative electron microscopy images of lysosomes (L) in dopaminergic neurons treated or not (CTRL) with 0.5 mM CBE for 29 days and stained with uranyl acetate and lead citrate. N, nucleus; M, mitochondrium and quantification of the number of lysosomes and their size. Data are expressed as fold change with respect to CBE-untreataed cells (CTRL) or as area value of CBE treated and untreated (CTRL) cells and are the mean ± SEM of at least three experiments; ** p < 0.01, Student's t-test vs CBE-untreated cells.

Supplementary figure S12. Evaluation of lysosomal catabolism of glycosphingolipids upon glucosylceramide accumulation. Representative HPTLC image and quantification of sphingolipids extracted from mouse cerebellar granule neurons treated or not with 0.5 mM conduritol B epoxide (CBE) for 14 days followed by radioactive GM1 administration (Cer=ceramide; GlcCer= glucosylceramide; LacCer= lactosylceramide; Gb3=globo-triaosylceramide; GM3=monosialodihexosylganglioside; GM1= monosialo). Data are expressed as fold change with respect to CBE untreated cells (dashed line) and are the mean ± SEM of the from at least three experiments; * p < 0.05, ** p < 0.01, *** p < 0.001, Student's t-test vs CBE-untreated cells.

Supplementary figure S13. Interactome of plasma membrane proteins isolated from CBE treated neurons. Representation of functional interactions between precipitated plasma membrane proteins exclusively present in DA neurons treated with 0.5 mM CBE compared to untreated neurons. Interactions were obtained by analysis with STRING.

Supplementary figure S14. Characterization of Detergent Resistant Membrane fractions. Detergent Resistant Membrane fractions (DRM) were isolated from human iPSCs-derived dopaminergic neurons treated or not with 0.5 mM conduritol B epoxide (CBE) for 29 days after metabolic labelling with [1-^{3}H]-sphingosine. Fractions were collected and either counted for radioactivity or subjected to lipid extraction or immunoblotting with anti-DRM-specific marker antibodies. **a** Representative image of the expression of protein markers in the fractions no. 5-6 and in fractions 10 -12 **b** Percentage distribution of radioactivity associated with sphingolipids in the sucrose-gradient density fractions. **c** Percentage distribution of sphingolipids (SL) and phosphatidylethanolamine (PE) in DRM and HD fractions of CBE- untreated (CTRL) and treated DA neurons

Supplementary figure S1

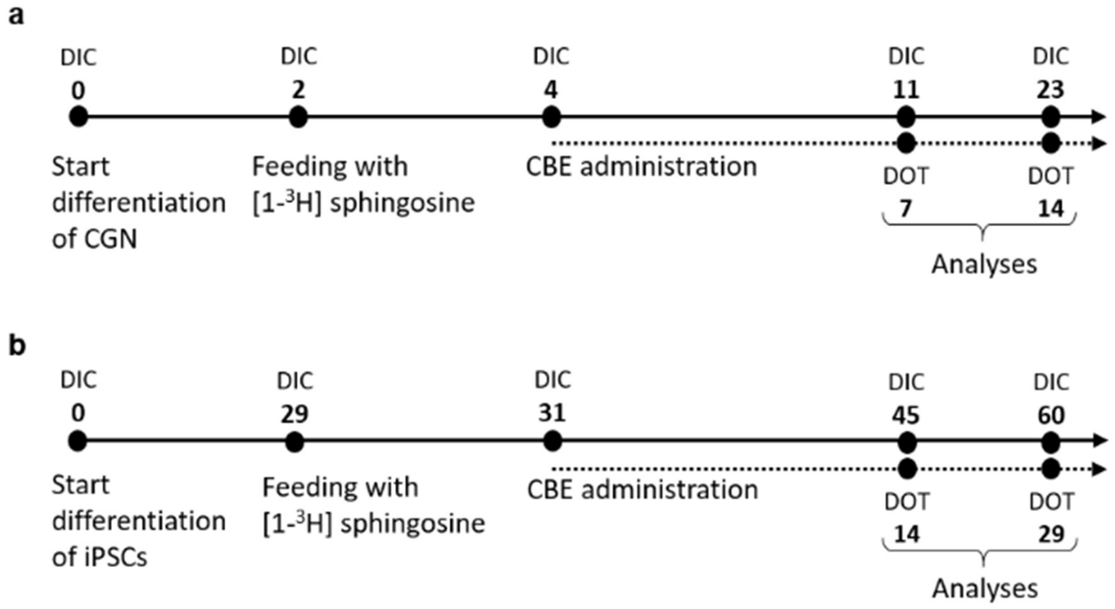

Supplementary figure S2

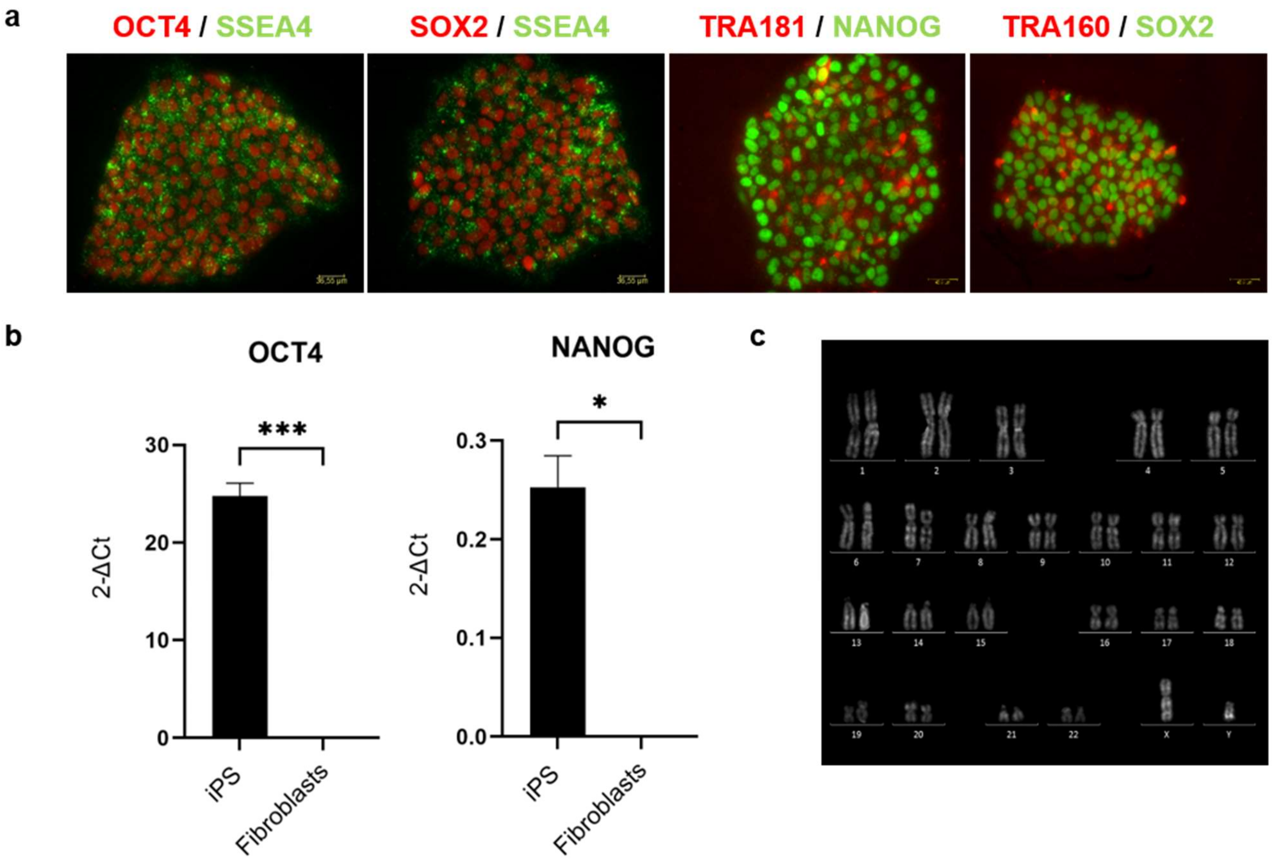

Supplementary figure S3

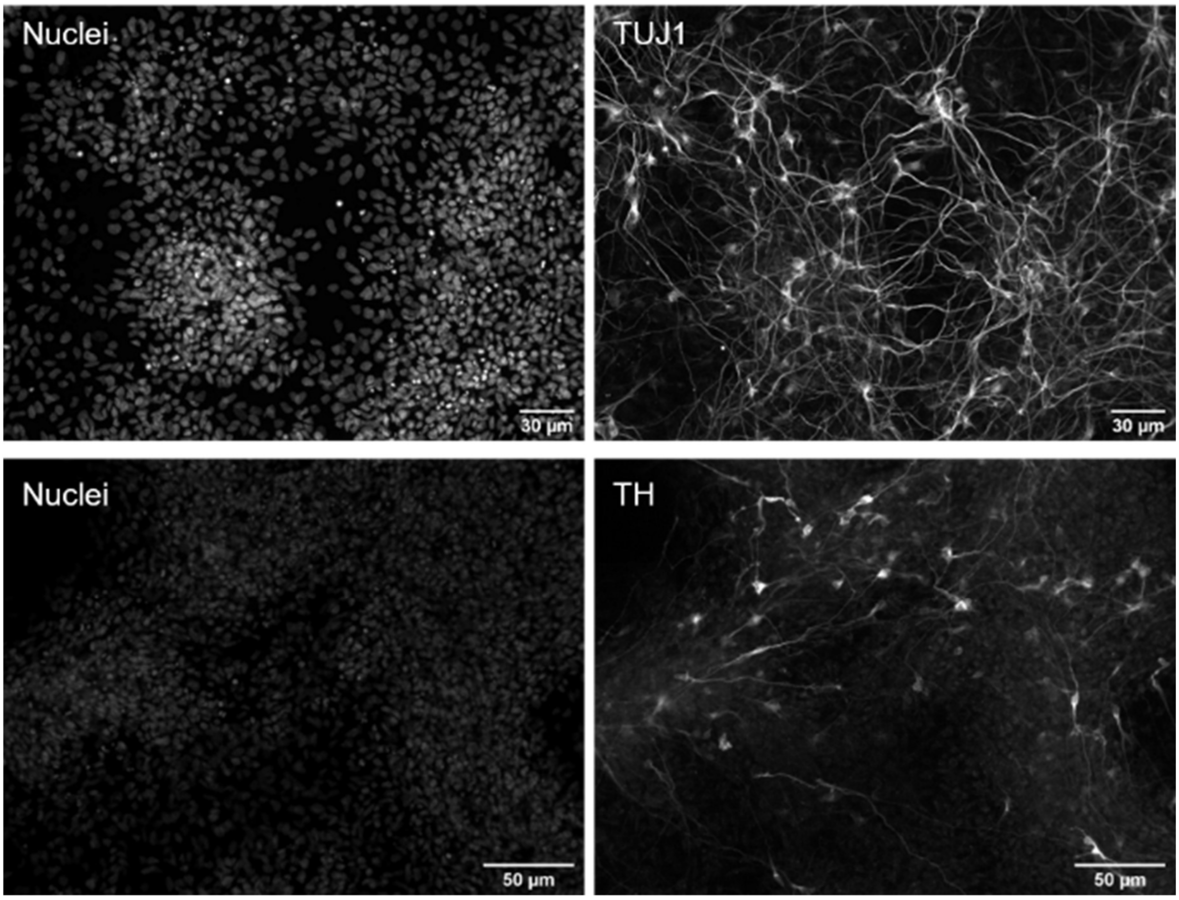

Supplementary figure S4

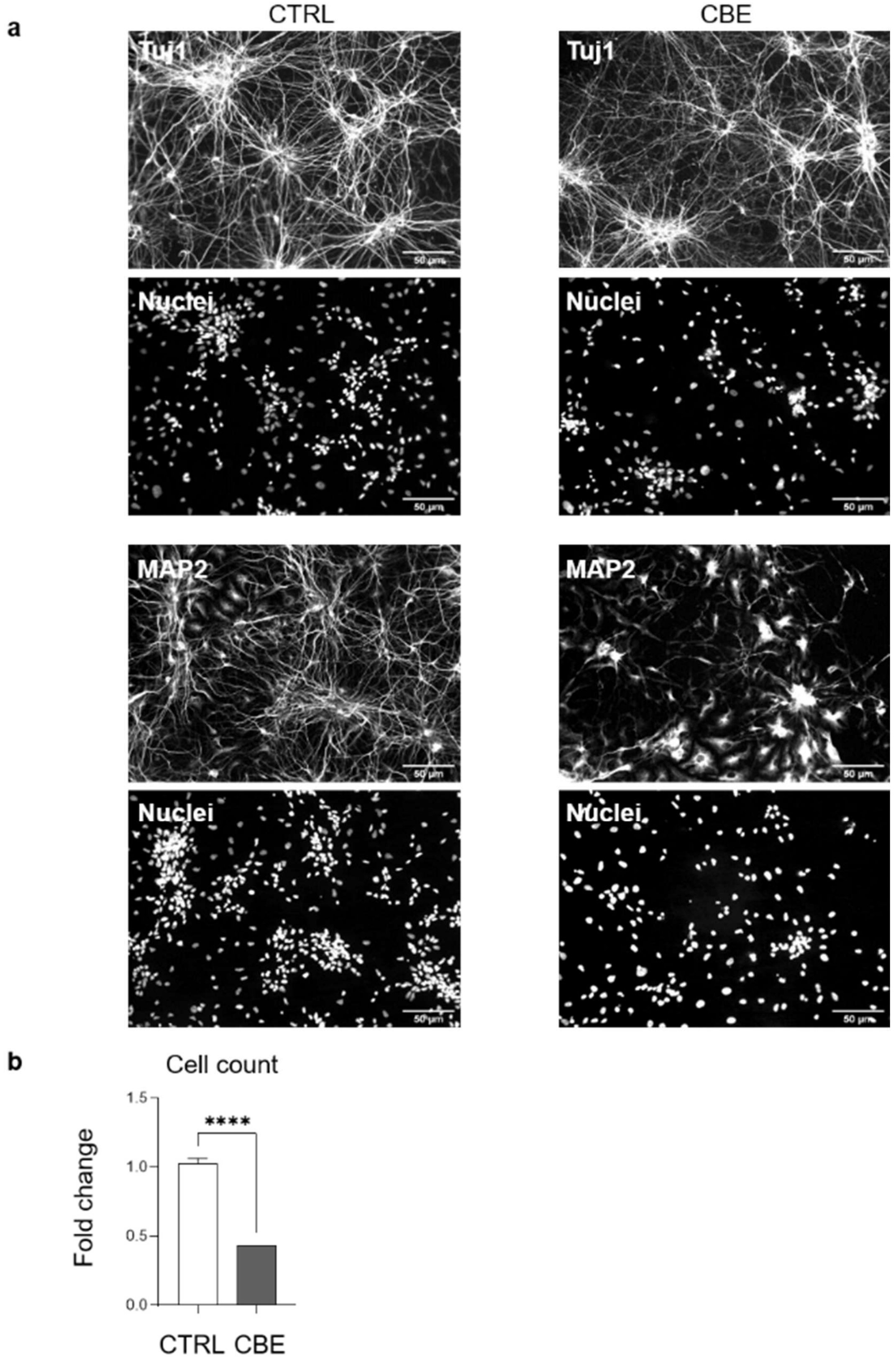

Supplementary figure S5

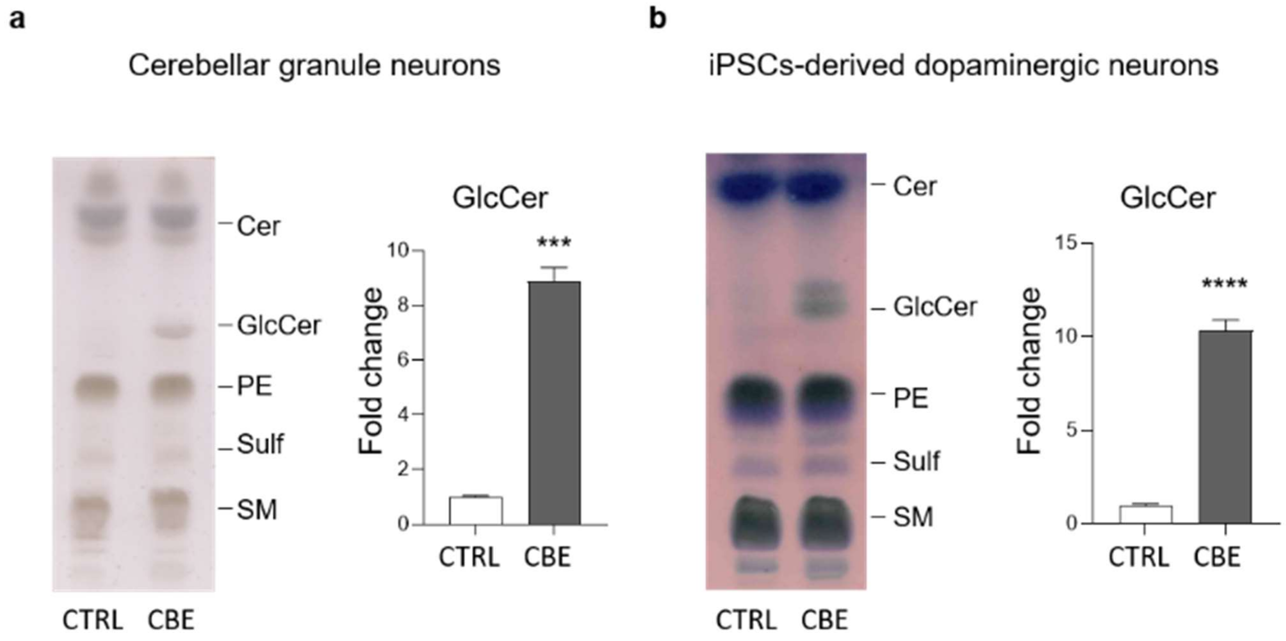

Supplementary figure S6

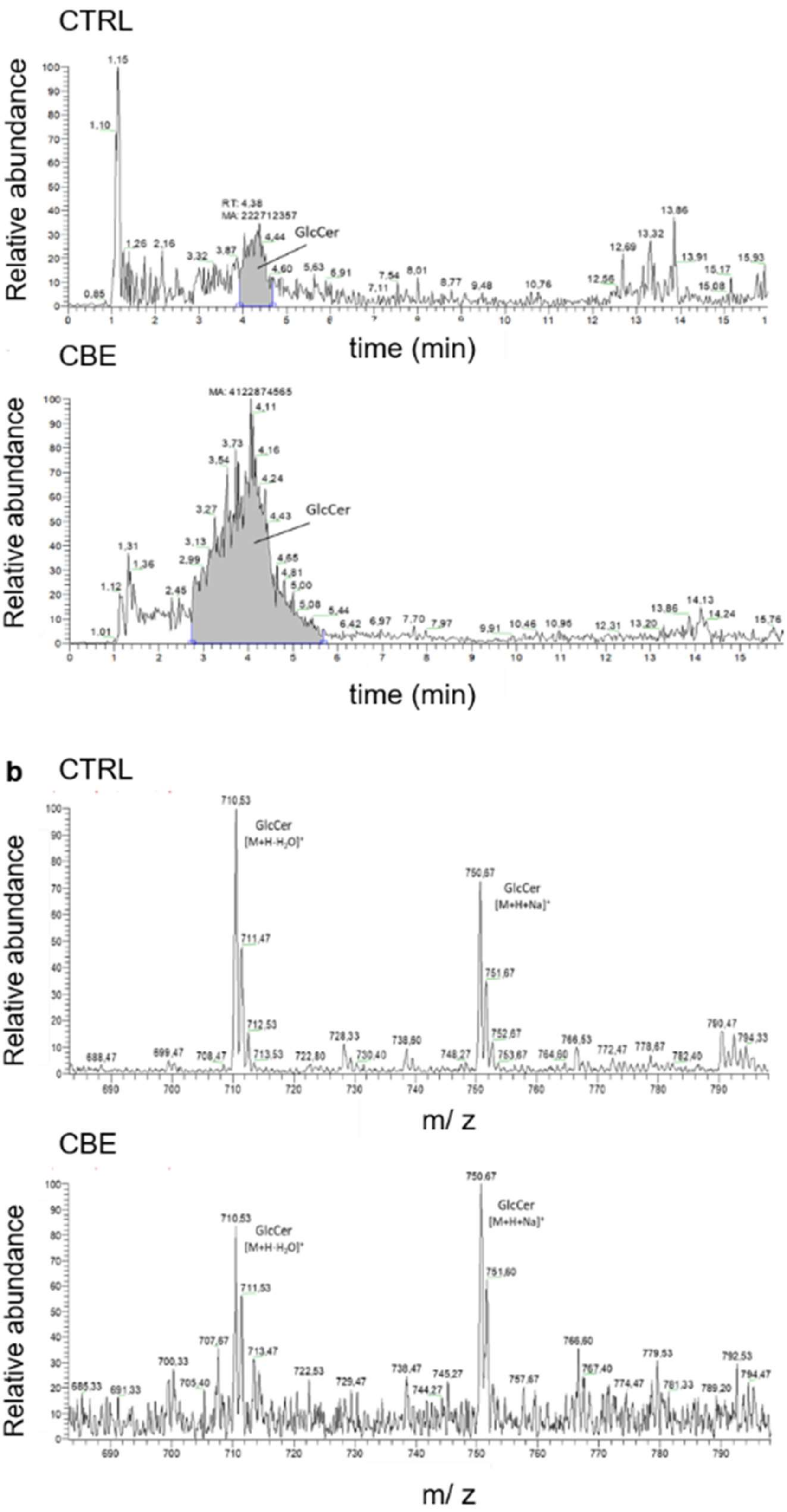

Supplementary figure S7

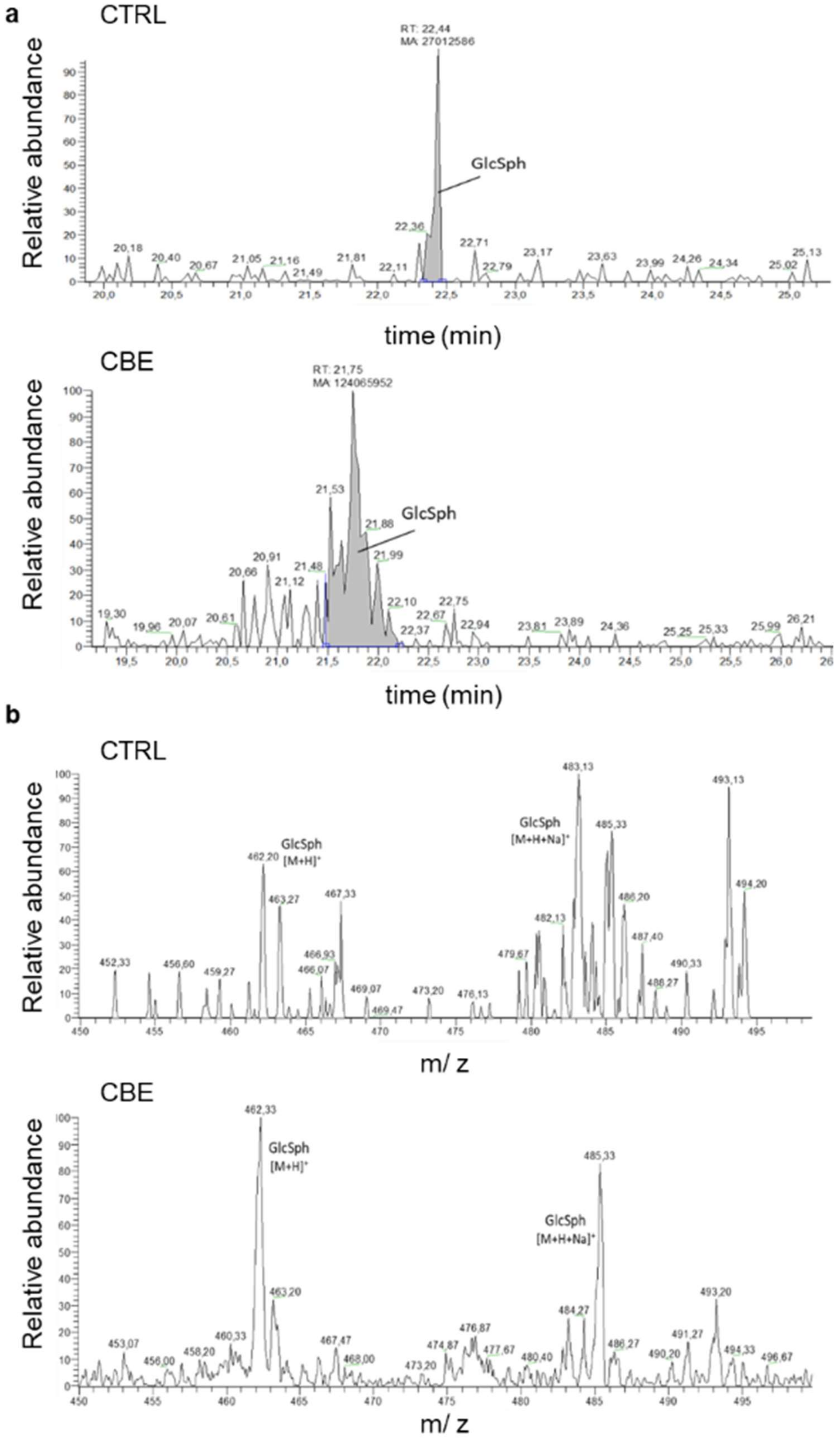

Supplementary figure S8

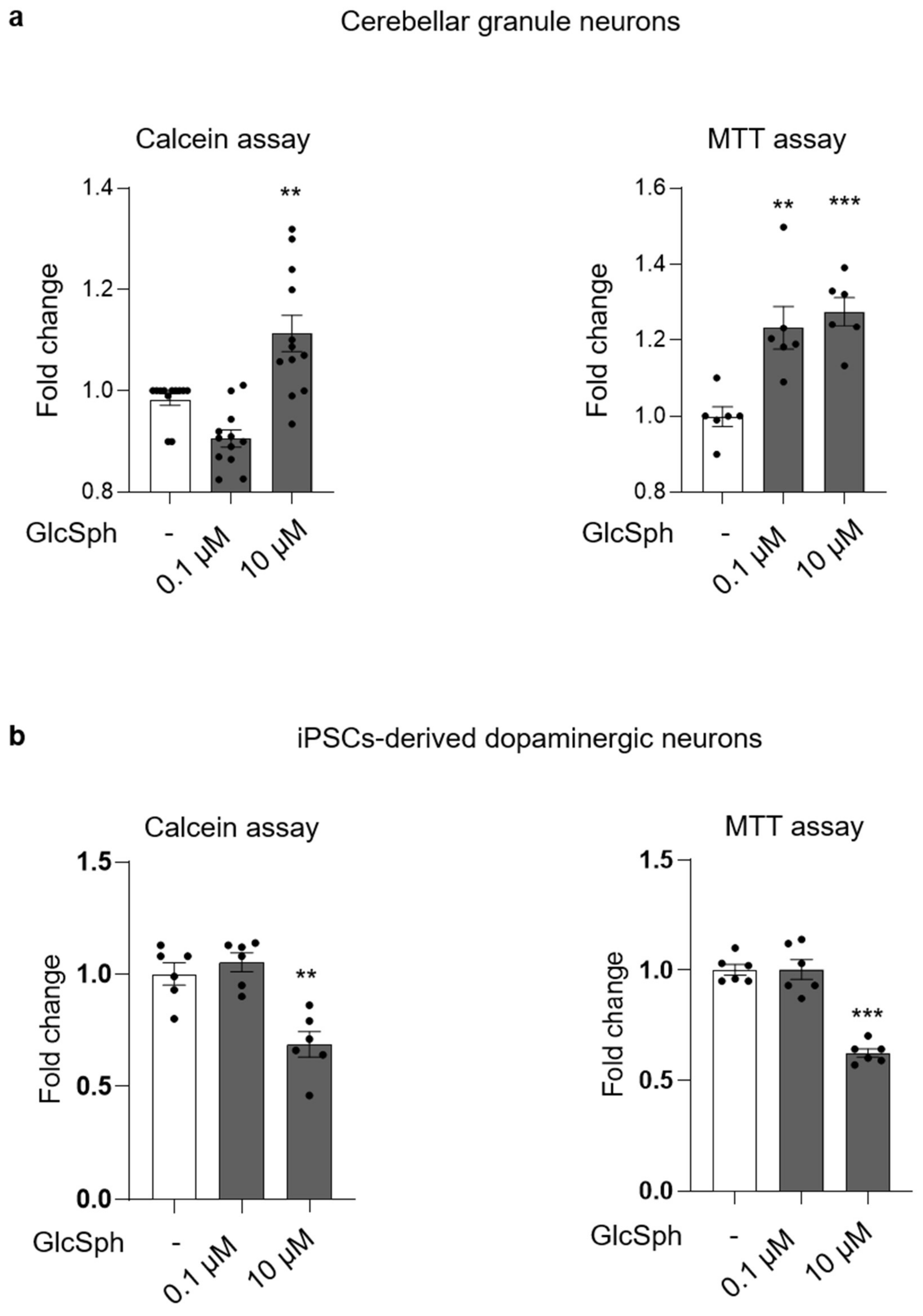

Supplementary figure S9

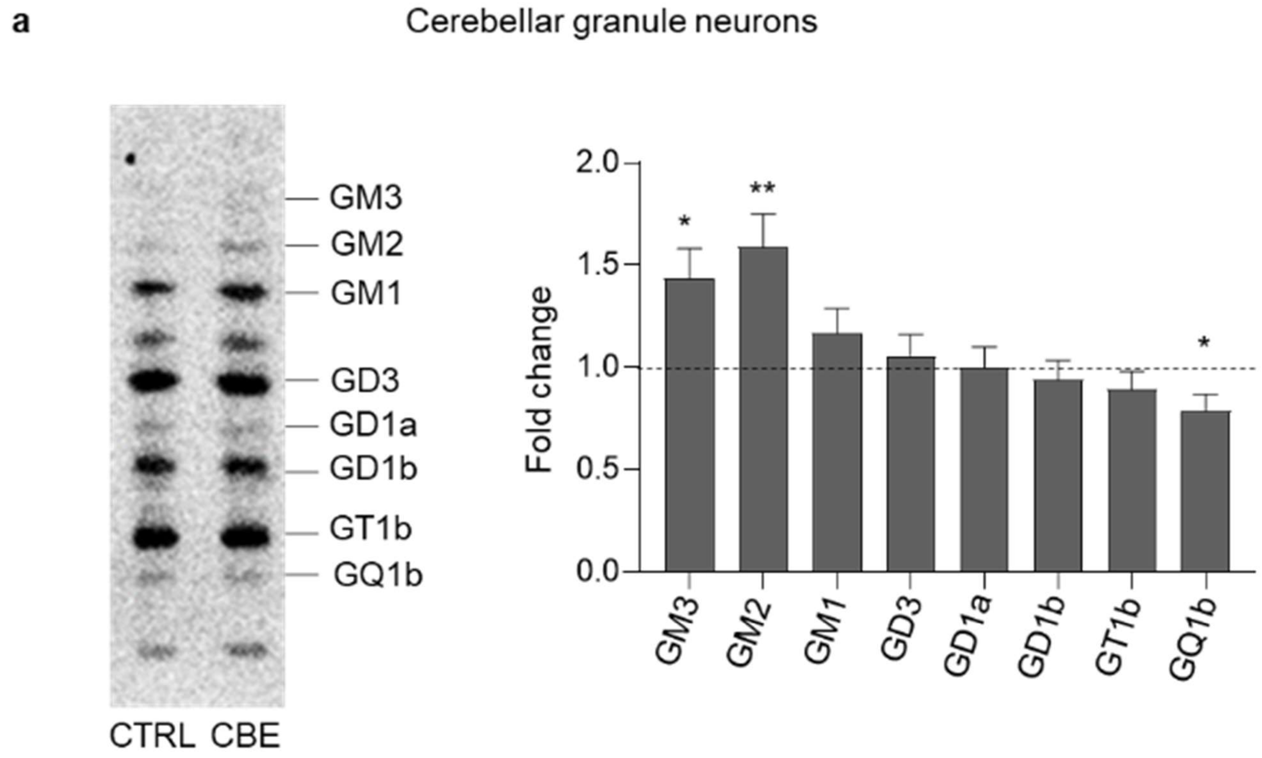

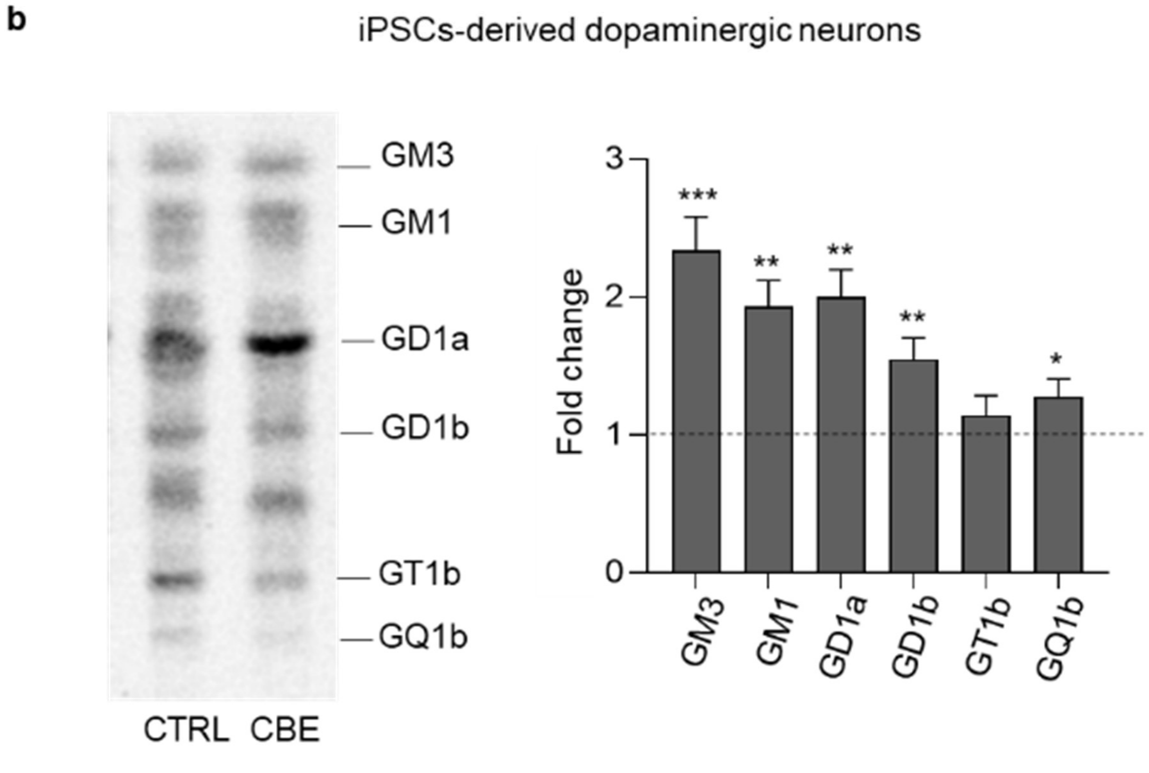

Supplementary figure S10

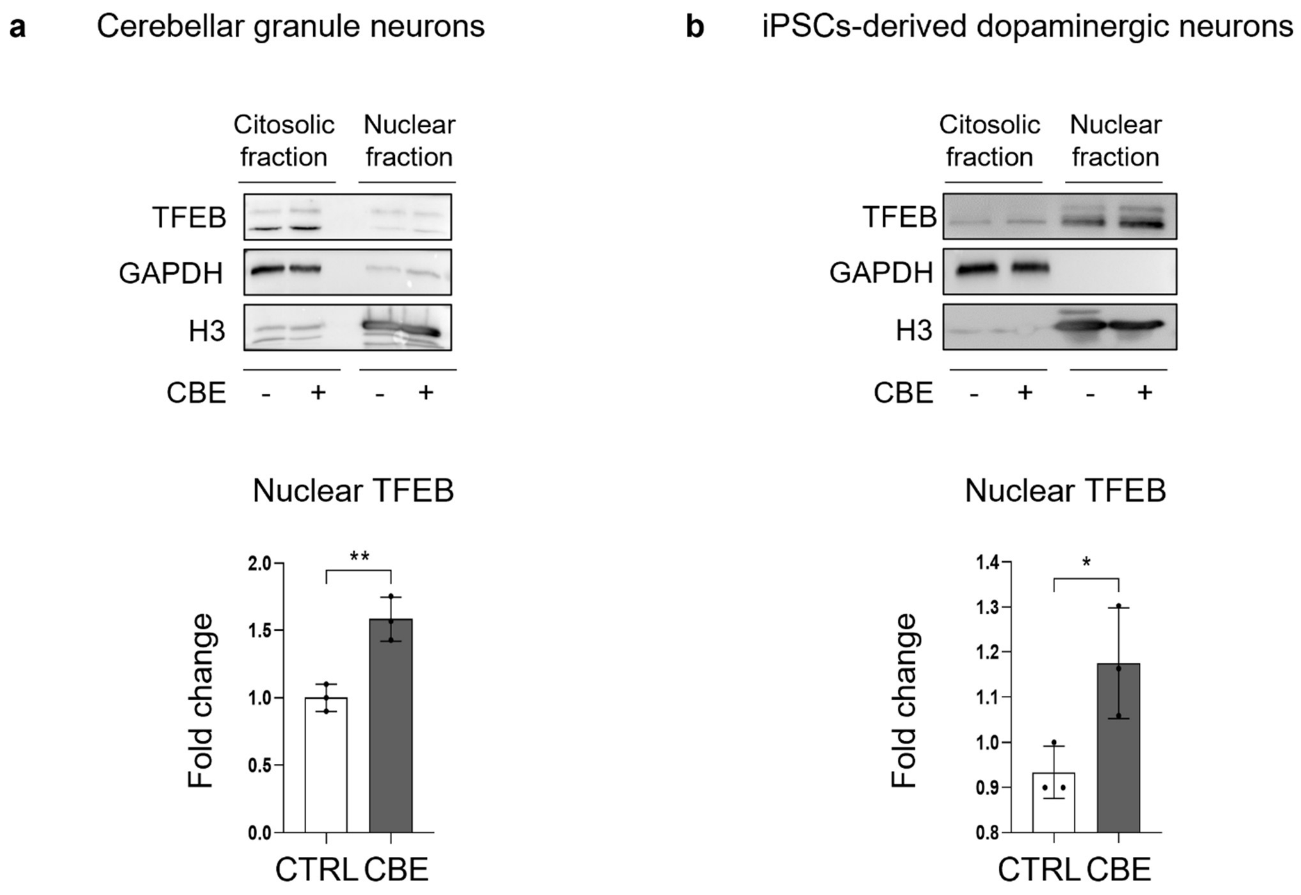

Supplementary figure S11

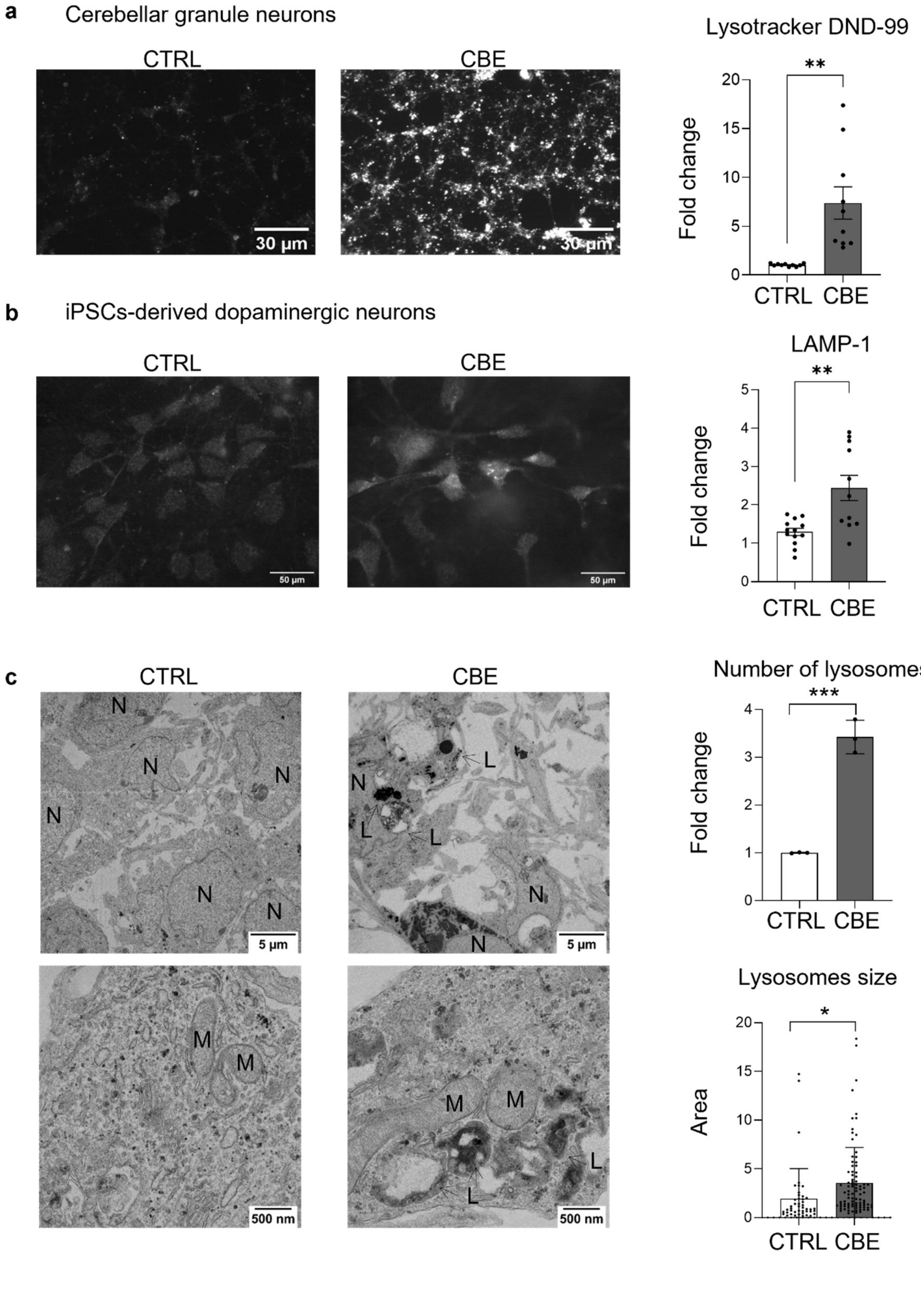

Supplementary figure S12

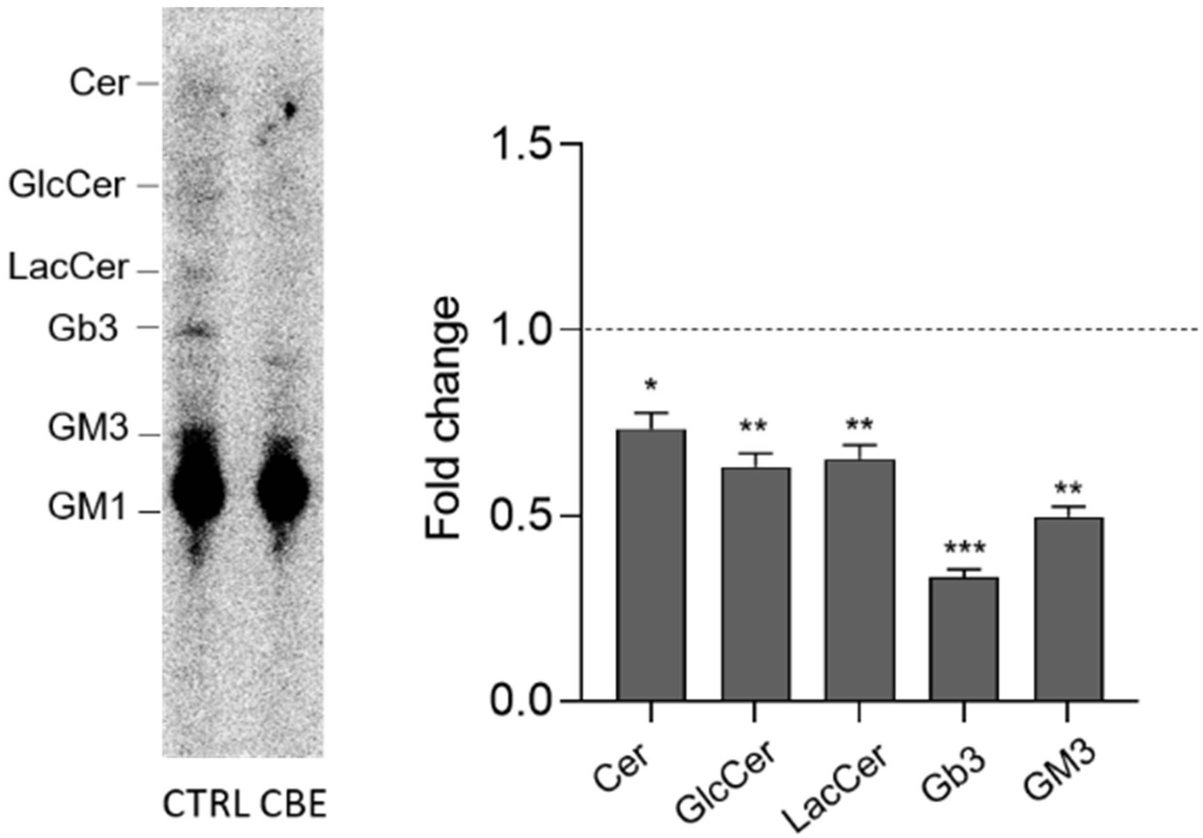

Supplementary figure S13

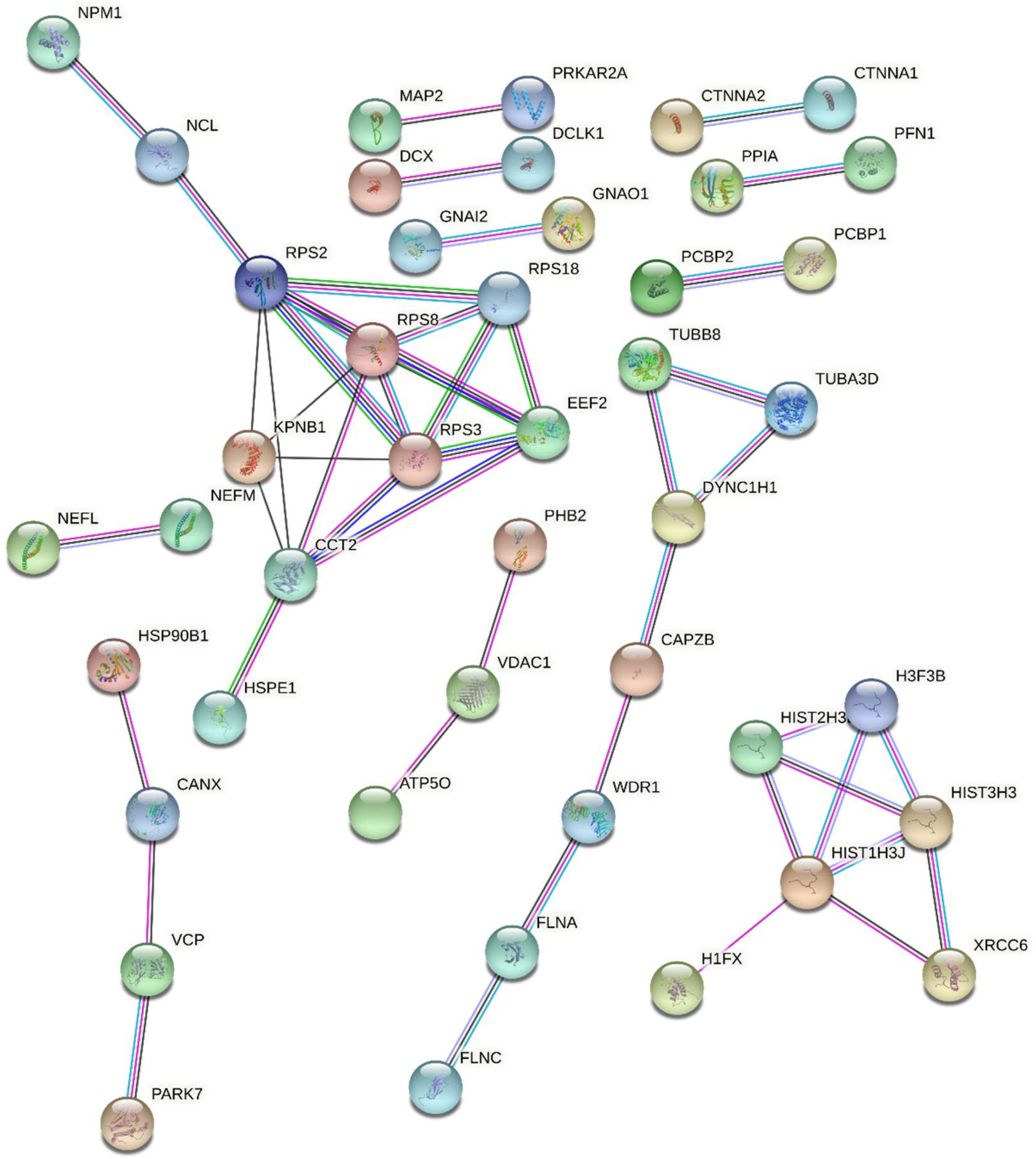

Supplementary figure S14

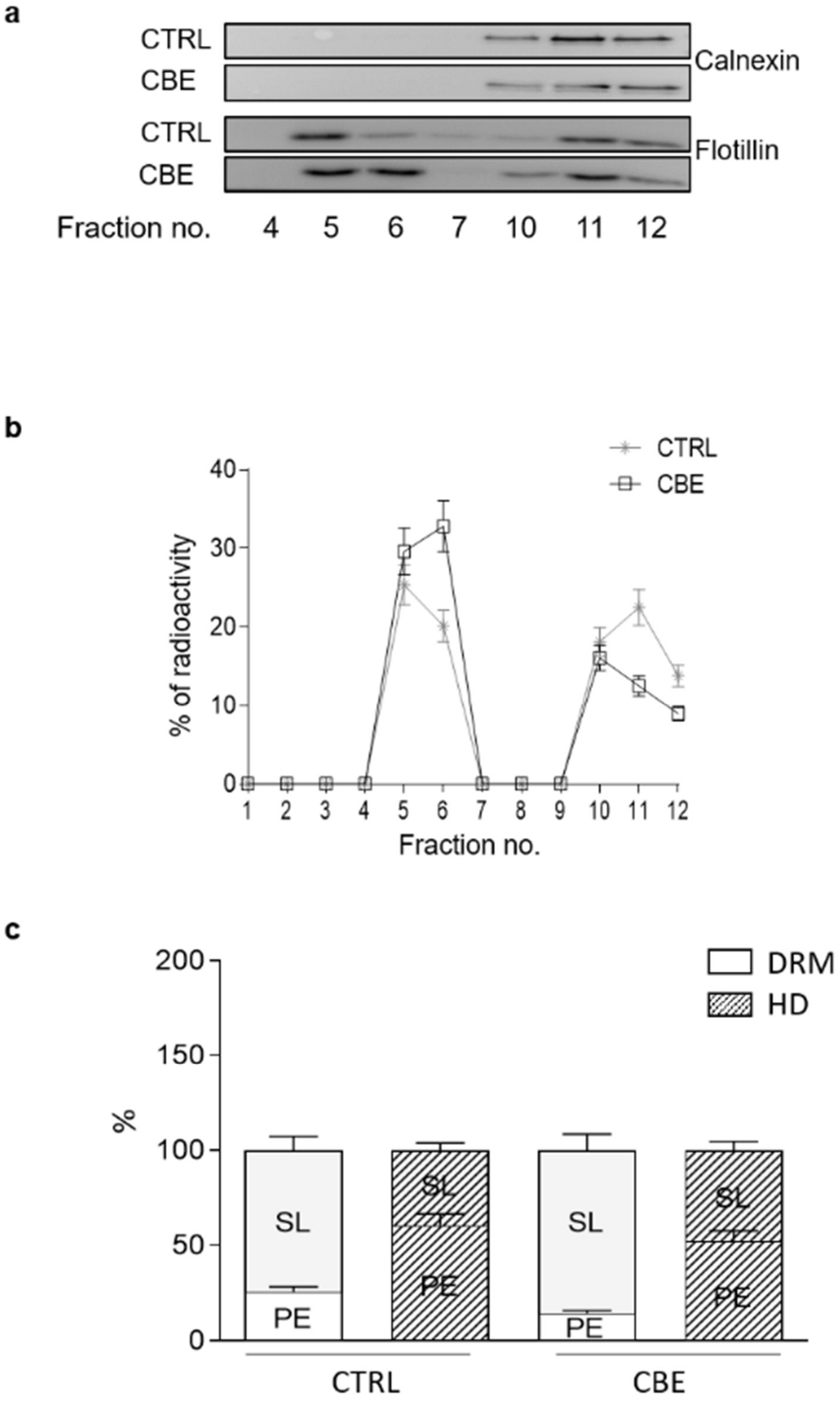

Supplementary table S1 List of the proteins upregulated in CBE-treated neurons compared to untreated cells.

ID	Gene name	Protein name	t-value	p-value
**P08962**	CD63	CD63 antigen	12,36	0,001
**P07602**	PSAP	Prosaposin (Sphingolipid activator protein)	11,84	0,000
**P17900**	SAP3	Ganglioside GM2 activator	10,33	0,000
**P78310**	CXAR	Coxsackievirus and adenovirus receptor	10,00	0,000
**Q9H0U6**	RM18	39S ribosomal protein L18, mitochondrial	7,31	0,001
**O00115**	DNS2A	Deoxyribonuclease-2-alpha	7,18	0,004
**P43003**	EAA1	Excitatory amino acid transporter 1	6,11	0,005
**P61916**	NPC2	NPC intracellular cholesterol transporter 2	5,46	0,010
**Q9C0H2**	TTYH3	Protein tweety homolog 3	5,42	0,012
**P06753**	TPM3	Tropomyosin alpha-3 chain	5,25	0,006
**Q9UK22**	FBX2	F-box only protein 2	5,19	0,006
**O15540**	FABP7	Fatty acid-binding protein, brain	5,11	0,004
**Q9NWB6**	ARGL1	Arginine and glutamate-rich protein 1	5,11	0,011
**Q9UPT8**	ZC3H4	Zinc finger CCCH domain-containing protein 4	5,06	0,007
**Q08722**	CD47	Leukocyte surface antigen CD47	4,69	0,006
**Q92889**	XPF	DNA repair endonuclease XPF	4,67	0,003
**P52943**	CRIP2	Cysteine-rich protein 2	4,63	0,007
**P12956**	XRCC6	X-ray repair cross-complementing protein 6	4,35	0,008
**Q92542**	NICA	Nicastrin	4,27	0,006
**P11279**	LAMP1	Lysosome-associated membrane glycoprotein 1 (LAMP-1)	4,26	0,009
**O15400**	STX7	Syntaxin-7	4,09	0,018
**Q16850**	CP51A	Lanosterol 14-alpha demethylase (LDM)	3,93	0,008
**Q9H910**	JUPI2	Jupiter microtubule associated homolog 2	3,91	0,008
**P51608**	MECP2	Methyl-CpG-binding protein 2 (MeCp-2 protein)	3,90	0,020
**P34913**	HYES	Bifunctional epoxide hydrolase 2	3,87	0,014
**P53778**	MK12	Mitogen-activated protein kinase 12 (MAP kinase 12)	3,84	0,010
**Q96B54**	ZN428	Zinc finger protein 428	3,80	0,028
**Q16352**	AINX	Alpha-internexin	3,71	0,020
**P28799**	GRN	Progranulin	3,69	0,027
**P46821**	MAP1B	Microtubule-associated protein 1B (MAP-1B)	3,63	0,033
**Q9UHG2**	PCS1N	ProSAAS (Proprotein convertase subtilisin/kexin type 1 inhibitor)	3,63	0,019
**Q9H1E5**	TMX4	Thioredoxin-related transmembrane protein 4	3,58	0,014
**Q14197**	ICT1	Peptidyl-tRNA hydrolase ICT1, mitochondrial	3,54	0,035
**O75781**	PALM	Paralemmin-1 (Paralemmin)	3,53	0,022
**P62263**	RS14	40S ribosomal protein S14	3,51	0,027
**Q16880**	CGT	2-hydroxyacylsphingosine 1-beta-galactosyltransferase	3,51	0,013
**B7ZBB8**	PP13G	Protein phosphatase 1 regulatory subunit 3G	3,49	0,021
**P54803**	GALC	Galactocerebrosidase	3,49	0,016
**P46976**	GLYG	Glycogenin-1	3,46	0,014
**Q6UWZ7**	ABRX1	BRCA1-A complex subunit Abraxas 1	3,45	0,033
**Q13185**	CBX3	Chromobox protein homolog 3	3,42	0,016
**P14209**	CD99	CD99 antigen	3,35	0,042

**O43657**	TSN6	Tetraspanin-6	3,23	0,019
**Q9P2W1**	HOP2	Homologous-pairing protein 2 homolog (Nuclear receptor coactivator GT198)	3,22	0,030
**Q01844**	EWS	RNA-binding protein EWS	3,20	0,023
**Q7Z4V5**	HDGR2	Hepatoma-derived growth factor-related protein 2	3,18	0,038
**P10620**	MGST1	Microsomal glutathione S-transferase 1	3,13	0,025
**Q9Y2B9**	IPKG	cAMP-dependent protein kinase inhibitor gamma (PKI-gamma)	3,09	0,023
**Q15262**	PTPRK	Receptor-type tyrosine-protein phosphatase kappa	3,08	0,045
**O94811**	TPPP	Tubulin polymerization-promoting protein (TPPP)	3,05	0,025
**Q14061**	COX17	Cytochrome c oxidase copper chaperone	3,05	0,033
**P20020**	AT2B1	Plasma membrane calcium-transporting ATPase 1	3,04	0,033
**Q8TB36**	GDAP1	Ganglioside-induced differentiation-associated protein 1 (GDAP1)	3,02	0,026
**O15347**	HMGB3	High mobility group protein B3	2,97	0,025
**Q969E2**	SCAM4	Secretory carrier-associated membrane protein 4	2,96	0,031
**P56211**	ARP19	cAMP-regulated phosphoprotein 19	2,95	0,027
**P37802**	TAGL2	Transgelin-2	2,92	0,031
**Q16799**	RTN1	Reticulon-1	2,91	0,046
**Q9NZ43**	USE1	Vesicle transport protein USE1 (Putative MAPK-activating protein PM26)	2,88	0,042
**Q9NS86**	LANC2	LanC-like protein 2	2,82	0,049
**Q9BWM7**	SFXN3	Sideroflexin-3	2,82	0,032
**Q6P1L5**	F117B	Protein FAM117B	2,80	0,033
**P61601**	NCALD	Neurocalcin-delta	2,69	0,046
**Q96BM9**	ARL8A	ADP-ribosylation factor-like protein 8A	2,67	0,048
**Q5SQI0**	ATAT	Alpha-tubulin N-acetyltransferase 1	2,63	0,040
**Q5T7N2**	LITD1	LINE-1 type transposase domain-containing protein 1	2,61	0,042
**Q9P2B2**	FPRP	Prostaglandin F2 receptor negative regulator	2,61	0,045
**Q9P0M9**	RM27	39S ribosomal protein L27, mitochondrial	2,60	0,044
**P18065**	IBP2	Insulin-like growth factor-binding protein 2 (IBP-2)	2,51	0,046
**Q8IVM0**	CCD50	Coiled-coil domain-containing protein 50	2,50	0,046

Supplementary table S2 List of the proteins down-regulated in CBE-treated neurons compared to untreated cells.

ID	Gene name	Protein name	t-value	p-value
**P26885**	FKBP2	Peptidyl-prolyl cis-trans isomerase FKBP2	-2,50	0,048
**Q9Y5L0**	TNPO3	Transportin-3 (Importin-12)	-2,54	0,045
**P62312**	LSM6	U6 snRNA-associated Sm-like protein LSm6	-2,56	0,046
**P29373**	RABP2	Cellular retinoic acid-binding protein 2	-2,64	0,040
**P62942**	FKB1A	Peptidyl-prolyl cis-trans isomerase FKBP1A	-2,64	0,039
**P56134**	ATPK	ATP synthase subunit f, mitochondrial	-2,68	0,037
**Q9BVT8**	TMUB1	Transmembrane and ubiquitin-like domain-containing protein 1	-2,69	0,048
**O15260**	SURF4	Surfeit locus protein 4	-2,71	0,047
**P61020**	RAB5B	Ras-related protein Rab-5B	-2,71	0,035
**P62861**	RS30	40S ribosomal protein S30	-2,74	0,040
**Q96EC8**	YIPF6	Protein YIPF6	-2,76	0,034
**Q9NR31**	SAR1A	GTP-binding protein SAR1a	-2,84	0,030
**Q14165**	MLEC	Malectin	-2,85	0,035
**O95861**	BPNT1	3'(2'),5'-bisphosphate nucleotidase 1	-2,86	0,044
**O43488**	ARK72	Aflatoxin B1 aldehyde reductase member 2	-2,87	0,029
**O43324**	MCA3	Eukaryotic translation elongation factor 1 epsilon-1	-2,93	0,043
**P38117**	ETFB	Electron transfer flavoprotein subunit beta	-2,99	0,025
**Q9NPJ3**	ACO13	Acyl-coenzyme A thioesterase 13	-3,02	0,027
**P51991**	ROA3	Heterogeneous nuclear ribonucleoprotein A3	-3,02	0,042
**P62310**	LSM3	U6 snRNA-associated Sm-like protein LSm3	-3,04	0,024
**P62318**	SMD3	Small nuclear ribonucleoprotein Sm D3	-3,05	0,030
**O94766**	B3GA3	Galactosylgalactosylxylosylprotein 3-beta-glucuronosyltransferase 3	-3,05	0,023
**P25208**	NFYB	Nuclear transcription factor Y subunit beta	-3,07	0,034
**Q9Y5U9**	IR3IP	Immediate early response 3-interacting protein 1	-3,10	0,024
**Q6BDS2**	URFB1	UHRF1-binding protein 1	-3,12	0,033
**P53597**	SUCA	Succinate--CoA ligase [ADP/GDP-forming] subunit alpha, mitochondrial	-3,17	0,028
**P61960**	UFM1	Ubiquitin-fold modifier 1	-3,17	0,020
**P22626**	ROA2	Heterogeneous nuclear ribonucleoproteins A2/B1	-3,20	0,026
**P60468**	SC61B	Protein transport protein Sec61 subunit beta	-3,21	0,025
**Q6NUQ4**	TM214	Transmembrane protein 214	-3,23	0,044
**Q16650**	TBR1	T-box brain protein 1	-3,34	0,030
**Q9H0V9**	LMA2L	VIP36-like protein	-3,43	0,039
**P24752**	THIL	Acetyl-CoA acetyltransferase, mitochondrial	-3,43	0,027
**P20340**	RAB6A	Ras-related protein Rab-6A (Rab-6)	-3,47	0,014
**P61081**	UBC12	NEDD8-conjugating enzyme Ubc12	-3,49	0,013
**P29762**	RABP1	Cellular retinoic acid-binding protein 1	-3,50	0,013
**O75396**	SC22B	Vesicle-trafficking protein SEC22b	-3,53	0,031
**Q96AY3**	FKB10	Peptidyl-prolyl cis-trans isomerase FKBP10	-3,60	0,034
**Q96EX1**	SIM12	Small integral membrane protein 12	-3,62	0,014
**Q9Y3D6**	FIS1	Mitochondrial fission 1 protein	-3,63	0,013
**Q5EB52**	MEST	Mesoderm-specific transcript homolog protein	-3,72	0,013

**O43772**	MCAT	Mitochondrial carnitine/acylcarnitine carrier protein	-3,74	0,012
**P61923**	COPZ1	Coatomer subunit zeta-1	-3,78	0,009
**O75880**	SCO1	Protein SCO1 homolog, mitochondrial	-3,85	0,019
**P09972**	ALDOC	Fructose-bisphosphate aldolase C	-3,86	0,026
**O60783**	RT14	28S ribosomal protein S14, mitochondrial	-3,98	0,007
**P13674**	P4HA1	Prolyl 4-hydroxylase subunit alpha-1	-4,00	0,010
**Q96DB5**	RMD1	Regulator of microtubule dynamics protein 1	-4,00	0,011
**Q07955**	SRSF1	Serine/arginine-rich splicing factor 1	-4,06	0,023
**Q5JWF2**	GNAS1	Guanine nucleotide-binding protein G(s) subunit alpha isoforms XLas	-4,10	0,011
**O43237**	DC1L2	Cytoplasmic dynein 1 light intermediate chain 2	-4,14	0,007
**O75323**	NIPS2	Protein NipSnap homolog 2	-4,45	0,011
**Q969M3**	YIPF5	Protein YIPF5 (Five-pass transmembrane protein localizing in the Golgi apparatus and the endoplasmic reticulum 5)	-4,48	0,004
**O75347**	TBCA	Tubulin-specific chaperone A	-4,50	0,007
**P32322**	P5CR1	Pyrroline-5-carboxylate reductase 1, mitochondrial	-4,57	0,005
**O60831**	PRAF2	PRA1 family protein 2	-4,58	0,015
**Q13596**	SNX1	Sorting nexin-1	-4,65	0,004
**P45877**	PPIC	Peptidyl-prolyl cis-trans isomerase C	-4,74	0,003
**Q9BUJ2**	HNRL1	Heterogeneous nuclear ribonucleoprotein U-like protein 1	-4,86	0,007
**Q969R2**	OSBP2	Oxysterol-binding protein 2	-5,06	0,002
**O14832**	PAHX	Phytanoyl-CoA dioxygenase, peroxisomal	-5,27	0,009
**Q9GZS3**	WDR61	WD repeat-containing protein 61	-5,75	0,002
**Q8TBQ9**	KISHA	Protein kish-A	-5,83	0,002
**Q71UI9**	H2AV	Histone H2A.V	-5,85	0,001
**O15269**	SPTC1	Serine palmitoyltransferase 1	-6,27	0,001
**Q96GQ5**	RUSF1	RUS family member 1	-6,46	0,001
**Q9HD45**	TM9S3	Transmembrane 9 superfamily member 3	-8,36	0,003
**Q13242**	SRSF9	Serine/arginine-rich splicing factor 9	-8,41	0,001
**P43307**	SSRA	Translocon-associated protein subunit alpha (TRAP-alpha)	-9,45	0,000
**Q9BRA2**	TXD17	Thioredoxin domain-containing protein 17	-9,89	0,000
**Q9Y5M8**	SRPRB	Signal recognition particle receptor subunit beta	-13,23	0,000

Supplementary table S3 List of the proteins only expressed in the IP of CBE treated neurons in the comparison CBE treated vs CBE untreated neurons

ID	Gene name	Protein name
**P61604**	CH10	10 kDa heat shock protein, mitochondrial
**P62269**	RS18	40S ribosomal protein S18
**P15880**	RS2	40S ribosomal protein S2
**P23396**	RS3	40S ribosomal protein S3
**P62241**	RS8	40S ribosomal protein S8
**P48047**	ATPO	ATP synthase subunit O, mitochondrial
**P27824**	CALX	Calnexin OS=Homo sapiens
**Q15417**	CNN3	Calponin-3 OS=Homo sapiens
**P13861**	KAP2	cAMP-dependent protein kinase type II-alpha regulatory subunit
**P35221**	CTNA1	Catenin alpha-1 OS=Homo sapiens
**P26232**	CTNA2	Catenin alpha-2 OS=Homo sapiens
**Q00610**	CLH1	Clathrin heavy chain 1 OS=Homo sapiens
**Q14204**	DYHC1	Cytoplasmic dynein 1 heavy chain 1
**P13639**	EF2	Elongation factor 2 OS=Homo sapiens
**P14625**	ENPL	Endoplasmin OS=Homo sapiens
**P47756**	CAPZB	F-actin-capping protein subunit beta
**P21333**	FLNA	Filamin-A OS=Homo sapiens
**Q14315**	FLNC	Filamin-C OS=Homo sapiens
**P00367**	DHE3	Glutamate dehydrogenase 1, mitochondrial
**P04899**	GNAI2	Guanine nucleotide-binding protein G(i) subunit alpha-2
**Q9UBI6**	GBG12	Guanine nucleotide-binding protein G(I)/G(S)/G(O) subunit gamma
**P09471**	GNAO	Guanine nucleotide-binding protein G(o) subunit alpha
**P16401**	H15	Histone H1.5
**Q92522**	H1X	Histone H1x
**P68431**	H31	Histone H3.1
**Q16695**	H31T	Histone H3.1t
**Q71DI3**	H32	Histone H3.2
**P84243**	H33	Histone H3.3
**P50502**	F10A1	Hsc70-interacting protein
**Q14974**	IMB1	Importin subunit beta-1
**Q9NZI8**	IF2B1	Insulin-like growth factor 2 mRNA-binding protein 1
**Q12906**	ILF3	Interleukin enhancer-binding factor 3
**Q9UK76**	JUPI1	Jupiter microtubule associated homolog 1
**P35908**	K22E	Keratin, type II cytoskeletal 2 epidermal
**P42166**	LAP2A	Lamina-associated polypeptide 2, isoform alpha
**P42167**	LAP2B	Lamina-associated polypeptide 2, isoforms beta/gamma
**P11137**	MTAP2	Microtubule-associated protein 2
**P60660**	MYL6	Myosin light polypeptide 6
**P29966**	MARCS	Myristoylated alanine-rich C-kinase substrate
**O95865**	DDAH2	N(G),N(G)-dimethylarginine dimethylaminohydrolase 2
**P13591**	NCAM1	Neural cell adhesion molecule 1

**P07196**	NFL	Neurofilament light polypeptide
**P07197**	NFM	Neurofilament medium polypeptide
**O43602**	DCX	Neuronal migration protein doublecortin
**P19338**	NUCL	Nucleolin
**P06748**	NPM	Nucleophosmin
**P62937**	PPIA	Peptidyl-prolyl cis-trans isomerase A
**Q06830**	PRDX1	Peroxiredoxin-1
**P30086**	PEBP1	Phosphatidylethanolamine-binding protein 1
**P18669**	PGAM1	Phosphoglycerate mutase 1
**Q15365**	PCBP1	Poly(rC)-binding protein 1
**Q15366**	PCBP2	Poly(rC)-binding protein 2
**Q92841**	DDX17	Probable ATP-dependent RNA helicase DDX17
**P07737**	PROF1	Profilin-1
**Q99623**	PHB2	Prohibitin-2
**Q99497**	PARK7	Protein/nucleic acid deglycase DJ-1
**P31150**	GDIA	Rab GDP dissociation inhibitor alpha
**O15075**	DCLK1	Serine/threonine-protein kinase DCLK1
**Q04837**	SSBP	Single-stranded DNA-binding protein, mitochondrial
**Q13813**	SPTN1	Spectrin alpha chain, non-erythrocytic 1
**Q7KZF4**	SND1	Staphylococcal nuclease domain-containing protein 1
**P78371**	TCPB	T-complex protein 1 subunit beta
**Q9UI15**	TAGL3	Transgelin-3
**P55072**	TERA	Transitional endoplasmic reticulum ATPase
**P29401**	TKT	Transketolase
**P55084**	ECHB	Trifunctional enzyme subunit beta, mitochondrial
**Q13748**	TBA3C	Tubulin alpha-3C/D chain
**Q3ZCM7**	TBB8	Tubulin beta-8 chain
**P09936**	UCHL1	Ubiquitin carboxyl-terminal hydrolase isozyme L1
**P21796**	VDAC1	Voltage-dependent anion-selective channel protein 1
**O75083**	WDR1	WD repeat-containing protein 1
**P12956**	XRCC6	X-ray repair cross-complementing protein 6

References

1 Valsecchi, M. *et al.* Ceramide and sphingomyelin species of fibroblasts and neurons in culture. *J Lipid Res* **48**, 417-424, doi:10.1194/jlr.M600344-JLR200 (2007).

2 Li, Y. T. *et al.* Selective extraction and effective separation of galactosylsphingosine (psychosine) and glucosylsphingosine from other glycosphingolipids in pathological tissue samples. *Neurochem Res* **36**, 1612-1622, doi:10.1007/s11064-010-0348-3 (2011).

3 Merrill, A. H., Jr. Sphingolipid and glycosphingolipid metabolic pathways in the era of sphingolipidomics. *Chem Rev* **111**, 6387-6422, doi:10.1021/cr2002917 (2011).

4 Shaner, R. L. *et al.* Quantitative analysis of sphingolipids for lipidomics using triple quadrupole and quadrupole linear ion trap mass spectrometers. *J Lipid Res* **50**, 1692-1707, doi:10.1194/jlr.D800051-JLR200 (2009).
